# Redox Regulation of cAMP-Dependent Protein Kinase and Its Role in Health and Disease

**DOI:** 10.3390/life15040655

**Published:** 2025-04-16

**Authors:** Ese S. Ekhator, Marco Fazzari, Robert H. Newman

**Affiliations:** 1 Department of Biology, North Carolina A&T State University, Greensboro, NC 27411, USA; ese33@pitt.edu; 2 Department of Pharmacology and Chemical Biology, University of Pittsburgh, Pittsburgh, PA 15261, USA; maf167@pitt.edu

**Keywords:** protein kinase, cAMP-dependent protein kinase (PKA), reactive oxygen species (ROS), oxidation, redox-dependent signaling, glutathionylation

## Abstract

Protein kinase A (PKA) is a key regulator of cellular signaling that regulates key physiological processes such as metabolism, cell proliferation, and neuronal function. While its activation by the second messenger 3′,5′-cyclic adenosine triphosphate (cAMP) is well characterized, recent research highlights additional regulatory mechanisms, particularly oxidative post-translational modifications, that influence PKA’s structure, activity, and substrate specificity. Both the regulatory and catalytic subunits of PKA are susceptible to redox modifications, which have been shown to play important roles in the regulation of key cellular functions, including cardiac contractility, lipid metabolism, and the immune response. Likewise, redox-dependent modulation of PKA signaling has been implicated in numerous diseases, including cardiovascular disorders, diabetes, and neurodegenerative conditions, making it a potential therapeutic target. However, the mechanisms of crosstalk between redox- and PKA-dependent signaling remain poorly understood. This review examines the structural and functional regulation of PKA, with a focus on redox-dependent modifications and their impact on PKA-dependent signaling. A deeper understanding of these mechanisms may provide new strategies for targeting oxidative stress in disease and restoring balanced PKA signaling in cells.

## 1. Introduction

Protein kinases, a family of enzymes that catalyze the transfer of the gamma (γ)-phosphate of ATP to specific phospho-acceptor sites (i.e., Ser, Thr, Tyr, or His residues) located on the surface of their target proteins, play a key role in most signal transduction cascades [1,2,3,4]. Kinase-mediated phosphorylation can affect the function of cellular proteins in diverse ways. For example, adding a negatively charged phosphate group to the surface of a protein can alter its cellular stability, enzymatic activity, protein–protein interactions, and/or subcellular localization [5,6]. Thus, phosphorylation-dependent signaling plays a vital role in regulating various biological processes, including the immune response, cell cycle progression, cell migration, differentiation, and metabolism [7,8,9,10,11]. Due to their central role in signal transduction, proper regulation of phosphorylation-dependent signaling pathways is critical to achieve normal cellular function [6]. Indeed, dysregulation of kinase activity leads to aberrant signaling that underlies the etiology and progression of many pervasive diseases, including diabetes, cancer, cardiovascular disease, and a variety of neurodegenerative disorders [4,12]. Similarly, dysregulation of cell signaling can impact pharmacological treatment outcomes [13]. Therefore, it is essential to understand how protein kinases, and the larger phosphorylation-dependent signaling networks of which they are part, are regulated inside the cell.

Over the past three decades, it has become clear that phosphorylation-dependent signaling networks are regulated by redox-dependent processes such as sulfenylation, disulfide bond formation, glutathionylation, and nitrosylation, both during normal physiological processes and in various disease states. For many years, it was believed that the primary point of crosstalk between redox- and phosphorylation-dependent signaling was at the level of protein tyrosine phosphatases (PTPs) [14]. However, an emerging body of evidence suggests that protein kinases can also be regulated by oxidation [15,16,17,18,19,20,21,22,23,24,25,26,27,28]. Unlike PTPs, which are almost universally inhibited by oxidation of an active site Cys residue involved in the catalysis of the phosphomonoester bond, the sites of oxidation in protein kinases are more diverse [17,18,20,22,23,24,25,26,29,30,31,32,33,34]. For instance, protein kinases have been shown to be oxidized on residues within their active sites, ligand binding regions, and regulatory domains. Likewise, the molecular consequences of oxidation are much more varied in kinases than in PTPs, with both oxidation-dependent increases and decreases in kinase activity having been reported (sometimes for the same kinase). Here, we explore some of the ways in which redox modification of the canonical protein kinase, 3′,5′-cyclic adenosine monophosphate (cAMP)-dependent protein kinase (PKA), affects its function, both in vitro and in cells. To this end, we conducted a thorough review of the literature to identify the ways in which oxidation impacts PKA’s activation, substrate selection, and cellular regulation (representative search terms used for the literature review are provided in Table S1). Below, we first briefly introduce PKA and some key principles of redox-dependent signaling before turning our attention to the impact of redox modification on PKA function. 

## 2. The cAMP-Dependent Protein Kinase (PKA)

As a founding member of the AGC family of kinases and one of the most well studied eukaryotic kinases, PKA has provided a wealth of information about the structure, function, and regulation of protein kinases [35,36,37,38,39,40]. PKA, which is canonically activated by the binding of intracellular cAMP to each of its two regulatory (R) subunits, regulates the activity of its protein substrates by phosphorylating serine or threonine residues on their surfaces [41,42]. PKA is expressed in many different types of cells, where it regulates cellular processes such as gene expression, cellular proliferation, apoptosis, and the metabolism of lipids and carbohydrates [43,44,45] (Table S2). PKA also plays an important role in the regulation of higher-order tissue functions, such as learning and memory in the brain, contraction force in skeletal muscles, and ciliary function and motility in the gastrointestinal tract [46,47,48,49]. Likewise, PKA affects blood vessel permeability and growth, hemostasis, and renin secretion by the kidneys to control blood pressure and cardiac function [50,51,52,53,54,55].

**Figure 1:**
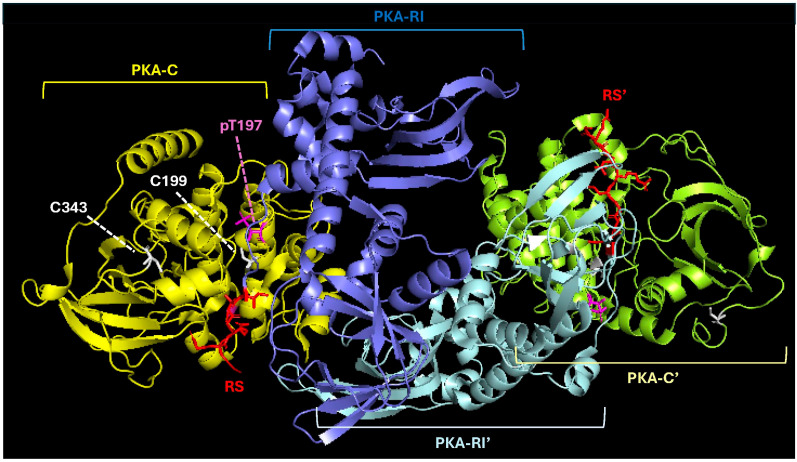
**Structure of the PKA holoenzyme.** The type I PKA holoenzyme comprises a homodimer of regulatory subunits (PKA-RI and PKA-RI’ shown in blue and silver, respectively) and two catalytic residues (PKA-C and PKA-C’ shown in gold and pale yellow, respectively). Each catalytic subunit associates with its cognate R subunit via a regulatory sequence (RS; red) in the regulatory subunit. The RS binds to PKA-C’s active site and holds it in an inactive state. The positions of PKA-C’s two Cys residues, C199 and C343 (white sticks), both of which are redox-sensitive, are shown. Likewise, the position of phospho-threonine 197 residue (pT197; magenta sticks) that plays a critical role in organizing PKA-C’s active site is shown. The PKA-RI subunits, which are held together by an N-terminal dimerization/docking (DD) domain that cannot be seen in the crystal structure, also each contain two redox-sensitive Cys residues, C17 and C38, in the DD domain (see Figure 2B for more details about the position of PKA-RI^C17^ and PKA-RI^C38^ in the DD domain). The image was created in PyMOL 3.1.3.1 based on PDB ID 6NO7 [61].

PKA exists as two primary types, type I and type II, each of which is a tetramer composed of two catalytic (C) and two R subunits [39,56,57,58] (Figure 1). The specific subtype (i.e., type I or type II PKA) is formally defined by the R subunits in the holoenzyme. For instance, while the type I PKA holoenzyme contains a homodimer of RI subunits, type II PKA contains two RII subunits [39,59]. Though RI/RII heterodimers can be formed in vitro, this configuration has not been observed in vivo [60].

**Figure 2:**
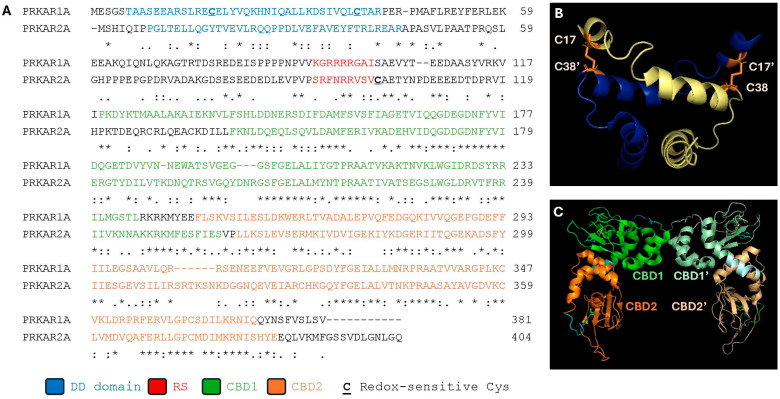
**Sequence homology and domain architecture of PKA regulatory subunits.** (**A**) Multiple sequence alignment of human PKA-RIα (PRKAR1A) and human PKA-RIIα (PRKAR2A) using Clustal-W. The dimerization/docking (DD) domain is shown in blue, the regulatory sequence (RS) is shown in red and the two cAMP binding domains, CBD1 and CBD2, are shown in green and orange, respectively. The redox-sensitive Cys residues in each isoform are underlined and in boldface. Identical residues are indicated with an asterisk, while highly similar and weakly similar residues are marked by ‘:’ and ‘.’, respectively. (**B**) The solution structure of the DD domain from bovine PKA-RIα showing the position of the redox-sensitive Cys residues, C17 and C38, in each strand. Note that C17 and C38 residues on opposite strands form an intermolecular disulfide bond. (**C**) Crystal structure of the bovine PKA-Riα homodimer highlighting the position of CBD1 (dark and light green in CBD1 and CBD1′, respectively) and CBD2 (dark and light orange in CBD2 and CBD2′, respectively). Structural images were created in PyMOL 3.1.3.1 using PDB ID 2EZW (**B**) and 4MX3 (**C**).

While different genes encode the RI and RII isotypes, the mRNA transcripts of each R-subunit gene can undergo alternative splicing to yield α and β isoforms [62]. Interestingly, a high degree of similarity is observed between the splice variants of RI and RII genes. For instance, the nucleotide sequences of the RIα and RIIα open reading frames are 75% identical at the nucleotide level, producing proteins with similar molecular weights that share 82% identity at the amino acid level [62,63] (Figure 2A). Regardless of the isoform, all PKA-R subunits share a similar architecture, consisting of a dimerization/docking (DD) domain, followed C-terminally by a PKA-C recognition sequence (RS) and two cAMP-binding domains (CBD A and B) (Figure 2B,C) [64,65]. Interactions between PKA-C and the PKA-R subunits hold PKA-C in an inactive state. While the RS present in each PKA-RII isoform contains a canonical PKA-C consensus motif ([R/K]-[R/K]-X-[S/T]) that is phosphorylated by PKA-C, the PKA-RI RS consists of a pseudosubstrate sequence in which an Ala (in the case of PKA-RIα) or a Gly residue (in the case of PKA-RIβ) has been substituted for the phospho-acceptor site [65,66]. As discussed in greater detail below, the regulatory subunits play an important role in the spatiotemporal regulation of the PKA holoenzyme, both with respect to its activation and its redox-dependent regulation. Despite their high similarity, the RI and RII isotypes are not functionally redundant. For instance, type I and type II PKA holoenzymes are targeted to different subcellular locales (and nanodomains) through interactions between their respective R subunits and specific A-kinase anchoring protein (AKAP) family members [67,68]. For example, AKAP2, AKAP6, and AKAP8 target type II PKA holoenzymes to the plasma membrane, nuclear membrane, and nucleus, respectively; meanwhile, the AKAPs ABCD3, SPHKAP, and MYO7A target type I PKA to the Golgi apparatus, mitochondria, and microtubules, respectively [69]. Interestingly, in addition to targeting the PKA holoenzyme to different subcellular regions, AKAPs and related scaffold proteins, such as A-kinase interacting proteins (AKIPs), bind distinct PKA substrates [70]. In so doing, they help regulate which PKA substrates are phosphorylated upon activation of the C subunit.

## 3. PKA-C Isoforms

Three isoforms of the PKA-C subunit exist in humans: PKA-Cα, PKA-Cβ, and PKA-Cγ [71]. In addition, PrkX and PrkY, which are encoded by the *PRKX* and *PRKY* genes located on the X and Y chromosomes, respectively, exhibit a high degree of sequence homology to the PKA-C isoforms [72]. PKA-Cα, which is encoded by the *PRKACA* gene and is believed to be the dominant isoform, is expressed in most tissues [57]. Meanwhile, PKA-Cβ, which is also expressed in diverse tissues, particularly the nervous system and immune system, is encoded by the *PRKACB* gene [73,74,75]. Finally, PKA-Cγ, which is encoded by the *PRKACG* gene, is most likely expressed only in the testis (though there is little evidence to suggest that the PKA-Cγ protein is translated or has any functional role in testis physiology, leading to the suggestion that *PRKACG* (along with *PRKY*) may exist as a retrotransposon in humans) [72,76,77,78]. Though PKA-Cβ, PKA-Cγ, PrkX, and PrkY each contain a conserved redox-sensitive Cys residue at the primary site of PKA-Cα oxidation (i.e., C199) that can undergo various types of redox modification (e.g., sulfenylation, sulfinylation, sulfonylation, disulfide bond formation, glutathionylation, nitrosylation, etc.), to date, most studies on the redox regulation of PKA-C have focused on the alpha isoform. Therefore, unless otherwise noted, we will focus our discussion on the redox regulation of PKA-Cα. However, it is important to note that many of the same mechanisms of crosstalk between redox- and PKA-dependent signaling may be at play for other PKA-C isoforms, as well.

The human *PRKACA* gene, which is approximately 26,000 nucleotides long, is found on chromosome 19 on the reverse strand at p13.1 [79]. *PRKACA* comprises ten exons, which encode a 351-amino-acid protein (40 kDa) corresponding to PKA-Cα1 [71,80]. Interestingly, human *PRKACA* can also undergo alternative splicing to yield the 348-residue PKA-Cα2 (also known as PKA-Cα short (Cαs)) and the 427-residue PKA-Cα3 variants, both of which contain the redox-sensitive Cys at a site corresponding to C199 in PKA-Cα1. While PKA-Cα1 is ubiquitously expressed throughout human tissue, PKACα2, which contains a different 5′-exon that initiates transcription at a different start codon, is primarily expressed in sperm cells [73,81,82,83,84]. *PRKACA* orthologs have been found in over 90 organisms, including humans, mice, zebrafish, *C. elegans*, and yeast [71,85]. Targeted deletion of this gene in mice results in growth retardation in the few animals that survive [86]. Similarly, PKA-Cα deficiency has also been associated with spinal neural tube abnormalities [87]. A double knockout of PKA-Cα and PKA-Cβ, on the other hand, or haploinsufficiency of PKA-Cβ in the context of a complete PKA-Cα knockout, is embryonically lethal [86]. Meanwhile, deletion of PKA-Cβ leads to phenotypically normal animals [86,88]. These seemingly contradictory findings support the hypothesis that there is a high degree of redundancy in the PKA-C subunit functions coupled with a complex utilization of PKA-C subunit isozymes in different tissues [59,89].

## 4. Activation of PKA

**Figure 3:**
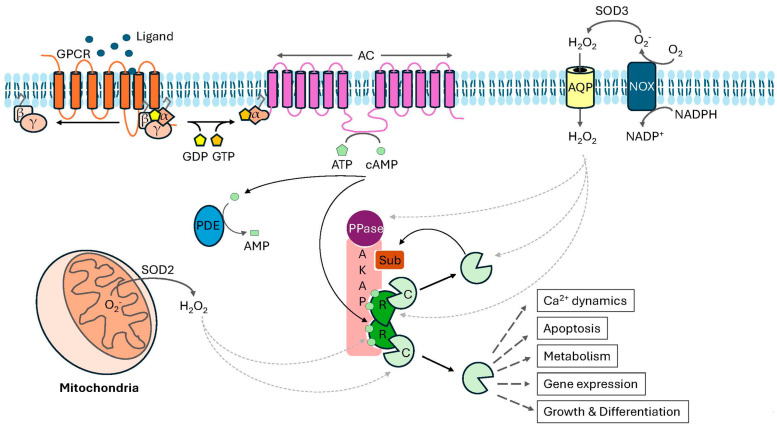
**Potential points of redox regulation during PKA-dependent signaling**. Schematic diagram illustrating the canonical 3′-5′-cyclic adenosine monophosphate (cAMP)-dependent activation of PKA (see text for details). Inefficient electron transport through complexes I and III in the mitochondria or activation of NADPH oxidase (NOX) enzymes in the plasma membrane lead to elevated hydrogen peroxide (H_2_O_2_) levels that can oxidize different components of the PKA signaling axis, including the PKA regulatory (R) and catalytic (C) subunits and regulatory proteins, such as protein phosphatases (see text for details). Abbreviations: GPCR: G protein-coupled receptor; α, β, γ: the small G proteins Gα_s_, Gβ, and Gγ; GDP: guanosine diphosphate; GTP: guanosine triphosphate; ATP: adenosine triphosphate; cAMP: cyclic adenosine monophosphate; AMP: adenosine monophosphate; AC: adenylyl cyclase; AKAP: A-kinase anchoring protein; Sub: PKA-specific substrate; PDE: phosphodiesterase; PPase: protein phosphatase; AQP: aquaporin; SOD: superoxide dismutase; O_2_: molecular oxygen; O_2_^−^: superoxide.

PKA activation begins with the binding of a ligand, such as the hormones epinephrine, prostaglandin E2 (PGE2), or glucagon, to a seven-transmembrane G protein-coupled receptor (GPCR) (Figure 3) [90,91]. The stimulatory G protein, known as Gα_s_, then activates membrane-bound adenylyl cyclase (AC) [92]. The ACs produce cAMP from ATP, leading to a >20-fold increase in the intracellular cAMP concentration seconds after stimulation [93,94]. Free cAMP binds to CBD B, causing a conformational change in the PKA-R subunits that exposes the CBD A [90,91]. This allows a second cAMP molecule to bind to each PKA-R subunit, inducing conformational changes that release the PKA-C subunits. Once activated, PKA-Cα can phosphorylate more than 380 cellular substrates, including transcription factors (e.g., cAMP-response element-binding protein (CREB), nuclear factor of activated T cells 4 (NFAT4)), other protein kinases (e.g., Fyn and Akt), and regulatory proteins (e.g., a regulator of G-protein signaling 14 (RGS14)), leading to numerous cellular outcomes, including regulation of metabolism, gene transcription, cell growth and division, and cell differentiation [95,96,97,98]. Therefore, dysregulation of PKA-dependent signaling contributes to the etiology and progression of many pervasive diseases, as discussed below.

## 5. PKA in Disease

Dysregulation of PKA activity has been implicated in the pathogenesis of numerous diseases, ranging from cancer and cardiovascular disease to metabolic and neurological disorders [62,99,100]. For instance, in the cardiovascular system, PKA signaling plays a crucial role in regulating cardiac contractility, vascular tone, and endothelial function [55]. Consequently, dysregulation of PKA-C subunits has been implicated in the pathophysiology of various cardiovascular disorders, including heart failure, arrhythmias, and hypertension. For example, altered expression and activity of PKA-Cα have been reported in failing hearts, contributing to impaired contractile function and pathological remodeling [43]. Additionally, dysregulated PKA signaling in vascular smooth muscle cells can lead to aberrant vasoconstriction and hypertension, highlighting the importance of maintaining proper PKA function for cardiovascular health [101,102,103].

Importantly, PKA is also a central regulator of cardiac contractile function, modulating intracellular Ca^2^⁺ handling and myofilament dynamics through phosphorylation of key substrates [104,105]. For example, studies have identified several PKA targets that contribute to the inotropic and lusitropic responses of cardiac myocytes, including phospholamban (PLN), the ryanodine receptor (RyR2), cardiac myosin-binding protein C (cMyBP-C), and cardiac troponin I (cTnI) [105,106,107,108,109]. Likewise, PKA-mediated phosphorylation of Rad, a Ras-like GTP-binding protein, at Ser25, Ser38, Ser272, and Ser300 relieves its inhibitory effect on the L-type Ca^2^⁺ channel (CaV1.2), thereby increasing channel open probability, enhancing Ca^2^⁺ influx, and contributing to the positive inotropic response [110]. In the sarcoplasmic reticulum (SR), PKA phosphorylates PLN at Ser16, disrupting its inhibitory interaction with the sarcoplasmic/endoplasmic reticulum Ca^2^⁺-ATPase (SERCA2) [111,112]. This leads to accelerated Ca^2^⁺ reuptake into the SR during diastole, facilitating myocardial relaxation and promoting positive lusitropy [105,106,107,108,113,114]. Additionally, PKA phosphorylation of RyR2 enhances Ca^2^⁺ release into the cytosol during systole, further strengthening contractility [109]. In the myofilament contractile machinery, PKA phosphorylates cMyBP-C at three sites within its cardiac-specific M-motif [115,116]. This modification releases the constraint of cMyBP-C on myosin S2 head domains, enhancing actin–myosin interactions and accelerating cross-bridge cycling, thereby contributing to increased contractile force [117,118]. Furthermore, phosphorylation of cTnI at Ser23/24 reduces sarcomere Ca^2^⁺ sensitivity by decreasing the affinity of the troponin C (cTnC) subunit for Ca^2^⁺, facilitating relaxation and improving lusitropy [119,120,121]. PKA-mediated phosphorylation of cMyBP-C and cTnI collectively reduces myofilament Ca^2^⁺ sensitivity, essential for efficient cardiac relaxation during diastole [122,123,124]. This Ca^2^⁺ desensitization is further supported by studies showing that phosphorylation of cTnI at Ser23/24 enhances the dissociation of Ca^2^⁺ from cTnC, thereby promoting rapid sarcomere relaxation [105,125,126,127].

Similarly, PKA signaling is intricately involved in regulating glucose and lipid metabolism, making it a key player in the development of metabolic disorders such as diabetes and obesity. Dysregulated PKA activity, particularly in adipose tissue and the liver, has been implicated in insulin resistance and dyslipidemia [128]. For instance, increased PKA-Cα activity in adipocytes has been associated with impaired insulin signaling and adipogenesis, contributing to insulin resistance and type 2 diabetes [129]. Similarly, dysregulated PKA signaling in hepatocytes can lead to abnormal glucose and lipid metabolism, exacerbating the progression of non-alcoholic fatty liver disease (NAFLD) and its complications [130]. Furthermore, genetic mutations in PKA-R subunits have been associated with rare metabolic disorders such as Carney syndrome, further underscoring the importance of PKA in metabolic homeostasis [131,132].

In the central nervous system, PKA signaling plays a critical role in synaptic plasticity, neuronal survival, and neurotransmitter release [133]. Dysregulated PKA activity has been implicated in various neurological disorders, including Alzheimer’s disease (AD), Parkinson’s disease (PD), and several mood disorders. For example, aberrant activation of PKA-Cγ has been linked to synaptic dysfunction and neurodegeneration in AD [134]. Additionally, dysregulated PKA signaling in dopaminergic neurons has been shown to participate in the pathogenesis of PD, highlighting the potential of therapeutic interventions targeting PKA-C in neurological disorders [135,136]. Similarly, altered PKA signaling in neurons can lead to synaptic dysfunction, neuronal apoptosis, and cognitive impairment, contributing to the pathogenesis of these disorders [137].

Finally, PKA signaling plays a complex role in cancer development and progression, with both tumor-promoting and tumor-suppressing effects observed depending on the cellular context [43,138]. Aberrant expression and activity of PKA catalytic subunits have been reported in various types of cancer. For instance, elevated levels of PKA-Cα are associated with increased cell proliferation, migration, and invasion in breast cancer [43,139,140]. Conversely, reduced expression of PKA-Cβ has been linked to tumor progression and metastasis in colorectal cancer [141]. Furthermore, mutations in the genes encoding the PKA-C and PKA-R subunits have been identified in certain malignancies, underscoring their importance in tumorigenesis [43,142]. Altered expression and activity of PKA-R subunits have also been reported in various types of cancer, where they play multifaceted roles in tumor development and progression. For instance, aberrant expression of PKA-RIα has been associated with increased cell proliferation and tumor growth in breast cancer [43,45,143]. Conversely, decreased expression of PKA-RIIβ has been linked to enhanced tumor invasiveness and metastasis in melanoma [43].

Understanding the molecular mechanisms underlying PKA dysregulation holds great promise for developing novel diagnostic and therapeutic strategies for these diseases. For instance, an emerging body of evidence suggests that there is extensive crosstalk between PKA- and redox-dependent signaling pathways in the etiology of many of these disorders. This crosstalk provides new insights into the regulation of PKA in both health and disease. Below, we first introduce some key concepts of redox-dependent signaling before outlining some of the ways in which redox modification impacts PKA-dependent signaling processes under both normal physiological conditions and in disease states.

## 6. Reactive Oxygen Species and Redox-Dependent Signaling

Reactive oxygen species (ROS) are a group of highly reactive oxygen derivatives consisting of radicals (e.g., superoxide (O_2_^−^) and hydroxyl radicals (•OH)) as well as non-radical peroxides (e.g., hydrogen peroxide (H_2_O_2_) and singlet oxygen) [15,144]. ROS are often generated in the mitochondria by inefficient electron transport during oxidative phosphorylation, primarily by complexes I and III of the electron transport chain [145,146]. However, over the past three decades, researchers have also come to appreciate the important role that regulated ROS production plays in normal cellular physiology and during the etiology and progression of various diseases [15,147,148,149,150,151,152,153,154,155,156,157,158,159,160,161,162]. For instance, aside from the incidental generation of ROS during cellular respiration, regulated ROS production by “professional” ROS-generating enzymes such as NADPH oxidase (NOX) family members is a critical step in redox-dependent signaling pathways [144,163,164,165,166,167,168]. Likewise, ROS are generated enzymatically by other important cellular enzymes, including glucose oxidase (GO), xanthine oxidase (XO), pyruvate dehydrogenase (PDH), and α-ketoglutarate dehydrogenase (KGDH) [169]. If left unchecked, rapid ROS production can lead to an intracellular environment characterized by oxidative stress. Indeed, excess ROS production contributes to the development and progression of various disease states, including cancer, diabetes, and cardiovascular disease [102,166,167,170,171,172,173,174,175]. For this reason, ROS were long thought to be exclusively detrimental to cellular systems; however, recent evidence suggests that they also act as essential signaling molecules in many normal physiological processes [15,19,27,62,148,149,151,173,176]. For instance, current research suggests that low levels of ROS contribute to the regulation of critical physiological processes, such as cell cycle progression, blood pressure regulation, cognitive processing, and immune function, both directly and indirectly [176,177,178,179,180,181,182,183,184].

Cells primarily produce three oxidants: H_2_O_2_, •OH, and O_2_^−^. Each is found at varying concentrations and has a distinct biological half-life. However, due to its relatively long half-life, H_2_O_2_ functions as the primary signaling molecule in most redox-dependent signaling processes [62,145,154,185]. H_2_O_2_ is formed through various cellular processes, including the enzyme-catalyzed one-electron reduction of molecular oxygen by NOX family members, followed by the rapid conversion of O_2_^−^ to H_2_O_2_ by superoxide dismutase (SOD) and inefficient electron transport by complexes I and III during cellular respiration [149,151,154,186,187,188]. Cells use this type of redox signaling to alter proteins and other macromolecules under physiological conditions.

## 7. Redox Modification of Proteins

Due to their abundance and relatively high-rate constants for oxidation reactions, proteins are the primary targets of ROS within the cell, accounting for approximately 67% of oxidation events [189,190]. In fact, some researchers have proposed that proteins evolved to function as a buffer system, absorbing oxidants and preventing oxidation-induced DNA mutations or lipid membrane oxidation and rupture [191,192,193,194,195]. This trade-off likely resulted in oxidation-dependent changes to proteins—including kinases—that may have been functionally harmless, harmful, or advantageous [15,196]. The implications of protein oxidation vary greatly from protein to protein, depending on their specific biochemical and structural features and the concentration and type of ROS involved. For example, oxidation-dependent changes in the charge, size, hydrophobicity, or polarity of amino acids can impact the secondary and tertiary structure of a protein, affecting its stability and function [15,154,196,197,198,199,200,201,202].

## 8. Cysteine Oxidation

**Figure 4:**
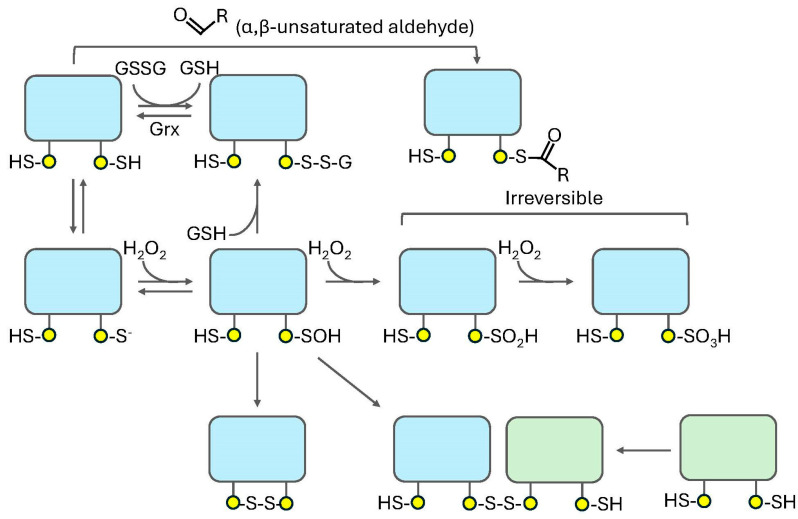
**Modes of Cys oxidation in proteins.** Cellular proteins undergo oxidation on Cys residues located on their surface (yellow circles). Redox-sensitive Cys residues, which exhibit a lower pK_a_ than standard sulfhydryl groups (Cys-SH), are first deprotonated to form a thiolate species (Cys-S^−^). In the presence of hydrogen peroxide (H_2_O_2_), the Cys-S^−^, which is a strong nucleophile, attacks the central O-O bond of H_2_O_2_ to generate a sulfenylated species (Cys-SOH). Cys-SOH, which is readily reversible to Cys-SH, can undergo hyperoxidation to sulfinylated (Cys-SO_2_H) and sulfonylated (Cys- SO_3_H) species that are largely irreversible inside the cell. To protect against hyperoxidation, Cys-SOH can also form either an intramolecular disulfide bond with another redox-sensitive Cys-SH residue in the same protein or an intermolecular disulfide with a redox-sensitive Cys-SH residue on another protein (green rectangle). These disulfide bonds can be reduced back to the basal state by the oxidoreductase, thioredoxin (Trx). Alternatively, the Cys-SOH can form a mixed disulfide by conjugation with small molecule antioxidants, such as reduced glutathione (GSH). A mixed disulfide can also be formed via disulfide exchange with oxidized glutathione (GSSG). The mixed disulfide can then be converted back to the basal state by the action of glutaredoxin (Grx). Finally, reactive Cys residues on proteins can be oxidized by secondary carbonylation (e.g., via conjugation to lipid peroxidation products of α,β-unsaturated aldehydes, such as malondialdehyde (MDA) or 4-hydroxynonenal (4-HNE)). Please see the text for additional details.

While several amino acids, such as methionine, histidine, tryptophan, and tyrosine residues, can be oxidized in cells, cysteine (Cys, C) is the most relevant amino acid with respect to redox-dependent signaling [196,199,203,204,205]. Indeed, among the 20 amino acids, Cys is the most functionally diverse and highly conserved [151,196,199,201,202,206]. The presence of basic amino acids in their local environment causes redox-sensitive Cys residues to exhibit a decreased pKa, leading to the conversion of the sulfhydryl (R-SH) moiety to a highly reactive thiolate species (R-S^−^) that is susceptible to oxidation by several cellular oxidizing agents, including H_2_O_2_ and nitric oxide (NO) [196,207,208,209] (Figure 4). Oxidation of R-S^−^ leads to both reversible and irreversible products. For instance, oxidation by H_2_O_2_ generates a sulfenic acid moiety (R-SOH) that is readily reversible back to the R-SH species. Further oxidation of the R-SOH moiety leads to the formation of sulfinic (R-SO_2_H) and sulfonic (R-SO_3_H) acid moieties, which are largely irreversible inside the cell [62,176,196]. Alternatively, to prevent hyperoxidation, R-SOH can form a covalent bond with another Cys residue within the same protein (to form an intramolecular disulfide bond), in another protein (to form an intermolecular disulfide bond), or on a small molecule reducing agent such as glutathione (to form a mixed disulfide bond) [151]. These disulfide bonds can be reduced back to the sulfhydryl species by members of the redoxin superfamily, such as glutaredoxin or thioredoxin [27,30,104,210,211,212,213,214,215]. Indeed, the ability of H_2_O_2_ to reversibly oxidize reactive Cys residues in cellular proteins under physiological conditions underlies its role in many signaling processes [91,154,199,201]. The consequences of H_2_O_2_-dependent oxidation vary greatly from protein to protein, depending on their specific biochemical and structural features. For example, as alluded to above, redox-dependent changes in the size, charge, hydrophobicity, or polarity of amino acids can alter the secondary, tertiary, and quaternary structure of the protein, impacting its stability, cellular localization, protein–ligand interactions, and enzymatic activity [15,196,199]. For instance, PKA family members have been shown to undergo H_2_O_2_-dependent oxidation on specific redox-sensitive Cys residues, leading to changes in their subcellular localization, interactions with regulatory factors, and substrate selection, all of which are described in the next section [27,62].

## 9. Redox Modification of PKA

Oxidative post-translational modifications (oxPTMs) such as sulfenylation, glutathionylation, and disulfide bond formation modulate PKA activity, leading to both activation and inhibition depending on the type of modification and the PKA sub-type under study. For instance, while oxidation of PKA-Cα by low concentrations of H_2_O_2_ has been shown to increase its activity toward several model substrates, diamide-dependent oxidation and/or glutathionylation leads to a marked decrease in its activity toward the same substrates [27,30]. Similarly, H_2_O_2_-dependent oxidation of type I PKA has been shown to enhance PKA-mediated phosphorylation of several cellular substrates, while oxidation of type II PKA leads to inhibition of its activity in cells [62,104]. The molecular bases for these differences are multifaceted and can provide important insights into the points of signal integration between redox- and PKA-dependent signaling pathways in health and disease.

## 10. Redox Regulation of Type I and Type II PKA Holoenzymes

**Figure 5:**
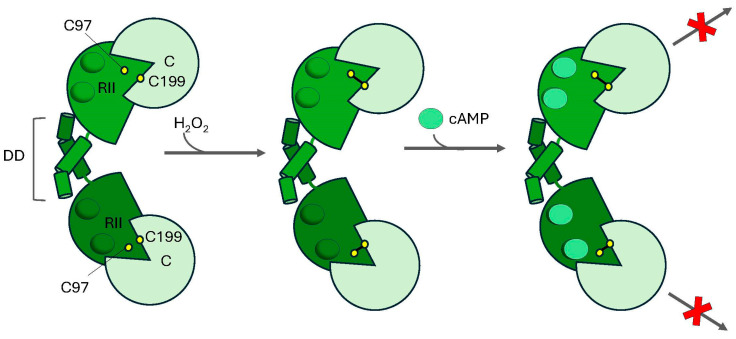
**Redox regulation of type II PKA.** In the presence of hydrogen peroxide (H_2_O_2_), redox-sensitive C199 residues in the catalytic subunits (C) form intermolecular disulfide bonds with C97 residues in the type II regulatory subunits (RII). Disulfide bond formation tethers the C subunit to RII, locking the holoenzyme in an inactive conformation even in the presence of cyclic AMP (cAMP; green circles), preventing the phosphorylation of downstream substrates (X).

In the case of the type II PKA holoenzyme, de Pina et al. found that signal-generated H_2_O_2_ following insulin stimulation in rat adipocytes led to the formation of an intermolecular disulfide bond between C199 in PKA-Cα (PKA-Cα^C199^) and C97 in PKA-RIIβ (PKA-RIIβ^C97^) [216]. Consequently, PKA-Cα remained sequestered in its inactive state, preventing lipolysis and failing to inhibit lipogenesis. This effect was abolished if the redox insensitive variant, PKA-Cα(C199A), was substituted for the wild-type PKA-Cα or if cAMP levels were increased prior to insulin addition. Together, these data suggest that, in the context of the type II holoenzyme, interactions between PKA-Cα^C199^ and PKA-RIIβ^C97^ are required for disulfide bond formation (Figure 5). These data are consistent with seminal studies by Susan Taylor’s laboratory who demonstrated that both PKA-Cα^C199^ and PKA-RII^βC97^ are susceptible to alkylation with iodacetic acid in isolation, but that they are shielded from modification in the ternary complex, suggesting that these residues are likely in close proximity to one another in the context of the holoenzyme [217]. However, due to the flexibility of residues 1–103 in the RIIβ protomer, PKA-RIIβ^C97^ is absent in the crystal structure of the type II holoenzyme [218]. Therefore, it is difficult to assess whether the holoenzyme must undergo a conformational change to properly align PKA-Cα^C199^ and PKA-RIIβ^C97^ to facilitate disulfide bond formation. Nonetheless, these studies suggest that PKA-Cα^C199^ and PKA-RII^C97^ come sufficiently close to one another in the holoenzyme to form a disulfide linkage. Interestingly, de Pina et al. also demonstrated that a similar mechanism may be at play in cells isolated from bovine heart, where pre-treatment of isolated cardiac cells with 1 μM H_2_O_2_ promoted an intermolecular crosslink between PKA-Cα^C199^ and PKA-RIIα^C97^, inhibiting PKA activity in these cells [216]. Thus, disulfide bond formation between the redox-sensitive PKA-Cα^C199^ and either PKA-RIIα^C97^ or PKA-RIIβ^C97^ prevents the release of the C subunit from the type II PKA holoenzyme, limiting its ability to phosphorylate its downstream substrates and attenuating PKA signaling [62].

In contrast, H_2_O_2_-dependent oxidation of type I PKA, which lacks a redox-sensitive residue analogous to C97 in the PKA-RII isoforms (Figure 2), has been found to promote the phosphorylation of several downstream PKA substrates in cells. Interestingly, this phenomenon appears to occur without observable changes in intracellular cAMP levels, suggesting an unconventional mode of PKA activation that is dependent on ROS [218,219]. For instance, Srinivasan et al. reported an increase in activated PKA-Cα in mitochondria following hypoxia in RAW 264.7 macrophages and during ischemia/reperfusion in an in vitro perfused mouse heart system [218]. ROS-induced activation of type I PKA led to phosphorylation and subsequent degradation of subunits I, IVi1, and Vb of complex IV of the electron transport chain, thereby attenuating its activity [218,220]. In this way, ROS-dependent activation of PKA is believed to prevent further ROS production during hypoxia/ischemia by feedback inhibition of complex IV [218,221]. H_2_O_2_-dependent activation of type I PKA was also recently shown to affect cardiac arrhythmias during periods of oxidative stress via a related mechanism. Specifically, Trum et al. demonstrated that type I PKA activated by elevated intracellular H_2_O_2_ levels extends the duration of action potentials in cardiomyocytes, leading to frequent “early afterdepolarizations” associated with many arrhythmias [222]. Similar H_2_O_2_-driven, cAMP-independent activation of type I PKA has also been implicated in several critical processes, including inflammation, angiogenesis, and Ca^2+^ handling during excitation–contraction–coupling in the myocardium [223,224].

**Figure 6:**
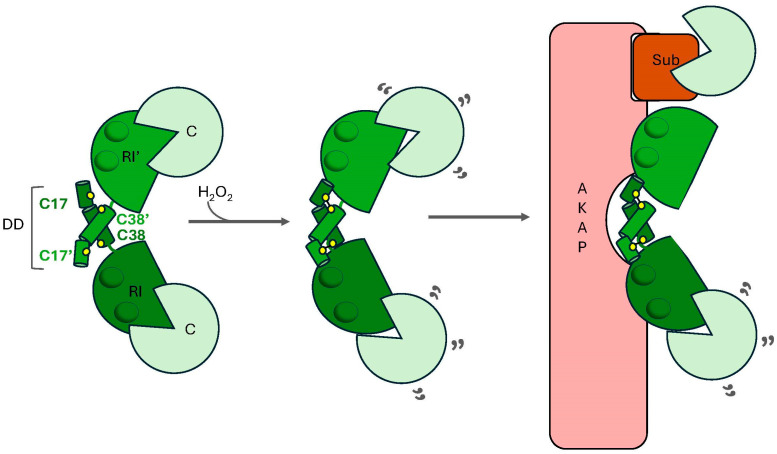
**Redox regulation of type I PKA.** In the presence of hydrogen peroxide (H_2_O_2_), residues located in the dimerization/docking domain (DD; green cylinders) of the regulatory subunits (PKA-RI; dark green hemisphere) of the type I PKA holoenzyme form an intermolecular disulfide bond that promotes the cAMP-independent activation of catalytic subunits (PKA-C; light green Pacman). Specifically, C17 in one PKA-RI monomer (RI) forms an intermolecular disulfide bond with C38 in the other PKA-RI monomer (RI’). Disulfide bond formation may destabilize interactions between the PKA-RI and PKA-C and/or promote interactions with specific A-kinase anchoring proteins (AKAP) in the cell. Association with AKAPs promotes the redistribution of the type I holoenzyme to discrete subcellular regions that bring PKA-C into close proximity to its substrates (Sub; red box) within a given nanodomain. The high local concentration of substrate may promote substrate-induced activation of PKA-C. Note that during the entire process, the cAMP binding domains (CBDs) in the PKA-RI subunits remain unoccupied.

ROS-induced activation of type I PKA is dependent on the formation of intermolecular disulfide bonds between C17 in one RI subunit and C38 in the adjacent RI subunit (Figure 6) [225]. These residues, which are located in the DD domain, lie anti-parallel to one another in the RI homodimer (Figure 2B) [226,227]. Notably, this was one of the first disulfide bonds ever discovered in an intracellular protein complex, offering early clues not only about the redox regulation of PKA but also about the existence of redox-dependent signaling pathways in cells [104,228]. While early models posited that the C17-C38 crosslink induces conformational changes in the PKA-RI homodimer that disrupt its interactions with bound PKA-C subunits, more recently, another intriguing hypothesis has emerged to explain the mechanism by which oxidation of type I PKA promotes the phosphorylation of distinct PKA substrates. Specifically, this theory holds that oxidation and the subsequent crosslinking of PKA-RI subunits promote interactions with select AKAPs, leading to the redistribution of the type I PKA holoenzyme to distinct subcellular regions where high local concentrations of specific PKA substrates promote substrate-induced activation of the kinase [62,219]. Several lines of evidence support this notion. First, as alluded to earlier, the C17-C38 crosslink is located within the DD domain, which mediates interactions between the RI homodimers and AKAPs [225]. In fact, the crystal structure of the ternary complex formed between the RI binding region of dual-specificity AKAP2 (D-AKAP2) and the PKA-RIα homodimer shows that each set of redox-sensitive residues flanks the core D-AKAP2 binding region [62,227]. Second, mutation of either C17 or C38 to Ala, which abolishes the regulatory subunit’s capacity to form the C17-C38 disulfide bond, causes a marked decrease in the affinity of RIα homodimers for D-AKAP2 [227]. Third, biochemical analyses suggest that high concentrations of substrate are necessary to fully activate type I PKA (but not type II PKA), lending support, at least theoretically, for the idea of substrate-induced activation of type I PKA [229]. Finally, researchers have observed oxidation-induced translocation of the type I PKA holoenzyme to various subcellular regions, including sarcomeres, the plasma membrane, the nucleus, and lysosomes [104,219,230]. For instance, Simon et al. recently demonstrated that ischemia/reperfusion (I/R) in cardiomyocytes obtained from both humans and mice led to an approximately two-fold increase in the formation of disulfide-linked dimers [230]. Oxidation of type I PKA promoted lysosomal targeting in an AKAP-dependent manner, where localized PKA prevented Ca^2+^ influx through two-pore channels (TPCs) located in lysosomes and limited I/R-induced Ca^2+^ overload. Importantly, substitution of a PKA-RIIα(C17S) variant with the wild-type protein in the context of a *PRKAR1A* knockout background abolished these effects and led to a significant increase in infarct size in PKA-RIIα(C17S)-expressing cells. These data suggest that oxidation of type I PKA protects cardiomyocytes from I/R-related injury by inhibiting TPC-mediated Ca^2+^ release and attenuating global intracellular Ca^2+^ influx from the sarcoplasmic reticulum. Of course, the AKAP model does not preclude a concomitant conformational change in RI that could destabilize or weaken the interaction between PKA-C and PKA-RI subunits in the holoenzyme, facilitating PKA-C release in the presence of high local concentrations of substrates and/or low basal concentrations of cAMP (Figure 6). Regardless, these studies support a model in which oxidation of type I PKA promotes the formation of disulfide bonds between C17 and C38, leading to interactions with cellular AKAPs that localize the kinase to specific subcellular compartments and nano/microdomains where their activation affects cellular physiology. Interestingly, the majority of known AKAPs specifically associate with the type II PKA holoenzyme, with only a handful of dual specificity and type I PKA-specific AKAPs having been described [69,231,232,233,234]. If oxidation of type I PKA promotes interactions with many of its scaffold proteins, the relatively low number of type I AKAPs discovered to date may be related to the experimental conditions used to screen for interactions (i.e., typically under reducing conditions). Therefore, in the future, it will be interesting to determine whether other AKAPs interact preferentially with oxidized type I PKA and how these interactions affect PKA function inside cells.

## 11. Redox-Dependent Modulation of PKA-C

**Figure 7:**
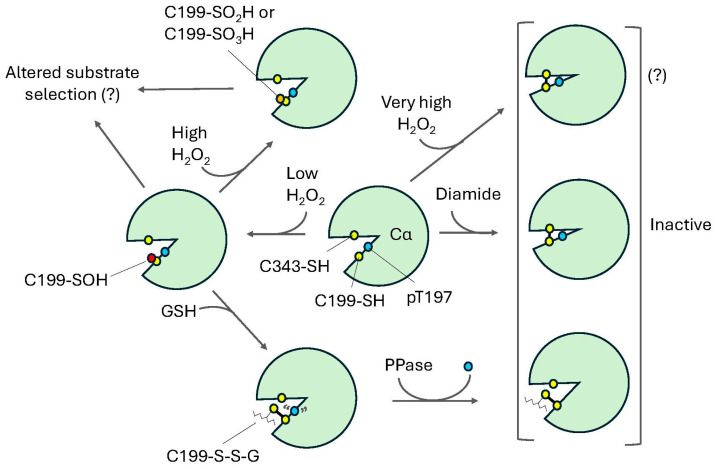
**Redox regulation of PKA-C.** PKA-Cα contains two redox-sensitive Cys residues, C199 and C343 (yellow circles), located in the P+1 loop and the large lobe, respectively. While C343 is far removed from the active site, C199 lies in close proximity to a critical phospho-Thr residue, pT197 (blue circle), in the activation segment. Treatment with low concentrations of hydrogen peroxide (H_2_O_2_) converts the more reactive C199 from the reduced form (C199-SH) to a sulfenylated species (C199-SOH). Sulfenylation of this residue has been shown to differentially alter the affinity of PKA-Cα for a series of model substrates, potentially leading to alternative substrate profiles in the oxidized and reduced states. Hyperoxidation of C199-SOH to either the sulfinylated (C199-SO_2_H) or the sulfonylated (C199-SO_3_H) species (orange circle) may contribute to further alterations in PKA-Cα’s substrate selection. Meanwhile, prolonged incubation with H_2_O_2_ or treatment with very high concentrations of H_2_O_2_ may promote the formation of an intramolecular disulfide bond between C199 and C343 that renders the kinase inactive. A similar intramolecular disulfide bond is observed following incubation of PKA-Cα with the chemical oxidizing agent diamide (a series of higher molecular weight species are also formed under these conditions, presumably due to the formation of intermolecular disulfide bonds between C199 and/or C343 on distinct molecules). Alternatively, sulfenylated PKA-Cα can be glutathionylated (C199-S-S-G) by reduced glutathione (GSH), an approach which may promote dephosphorylation of pT197, leading to kinase inactivation. The (?) indicates that, to our knowledge, direct observation of disulfide bond formation at very high H_2_O_2_ concentrations has not been experimentally verified, thus this relationship should be considered speculative at this time.

In addition, redox modification of PKA-Cα can directly affect its activity in several interesting ways. Importantly, once it is released from the holoenzyme, these effects do not appear to depend on PKA-Cα’s interactions with the R subunits. For instance, aside from PKA-Cα^C199^, which is located in the P+1 substrate binding loop within the activation segment, PKA-Cα also contains one other Cys residue, C343, which is found in the C-terminal tail region within the large lobe (Figure 2) [65]. While both residues can undergo redox modification, C199 appears to be more susceptible to oxidation than C343. Biochemical analysis suggests that, in addition to intermolecular disulfide bonds, these residues can also form intramolecular crosslinks (Figure 7). For instance, following treatment of purified PKA-Cα with the chemical oxidizing agent, diamide, native PAGE revealed several high molecular weight bands (presumably due to intermolecular disulfide bonds between C199 and/or C343 residues on different molecules) as well as a faster migrating species indicative of an internal disulfide bond (presumably between C199 and C343 on the same molecule) [27,30]. These effects were abolished by the addition of the reducing agent dithiothreitol (DTT) or by mutation of C199 to either Ala or Ser (PKA-Cα(C199A) and PKA-Cα(C199S), respectively), suggesting that disulfide bond formation is dependent on the oxidation of C199. The fact that C199 and C343, which are ~20 Å apart in a crystal structure of murine PKA-Cα (PDB ID: 1ATP), are able to form an intramolecular disulfide bond in the first place is somewhat surprising and may suggest that considerable flexibility exists within one or both of these regions of the catalytic subunit. This is consistent with molecular dynamics simulations of PKA-Cα conformers which revealed that PKA-Cα’s C-terminal tail is among the most flexible regions in the molecule [235].

Importantly, diamide-dependent oxidation of PKA-Cα led to a marked decrease in its activity toward the model peptide substrates, Kemptide and CREBtide [27,30]. Interestingly, the extent of inhibition was much more modest for another related PKA-Cα substrate, Crosstide [27]. While the phosphosites in both Kemptide and CREBtide are surrounded by residues that conform to the canonical PKA consensus phosphorylation motif ([R/K]-[R/K]-X-S/T), Crosstide contains a looser PKA consensus motif that lacks a basic residue at the P-3 position (i.e., three residues N-terminal to the phosphosite) [59,236]. This difference may render Crosstide less susceptible to conformational changes caused by diamide-mediated disulfide bond formation that otherwise disrupt PKA-Cα’s interactions with basic residues in the P-3 site in Kemptide and CREBtide.

Perhaps surprisingly, a similar change in PKA-Cα’s migration pattern by native PAGE was not observed when PKA-Cα was pre-treated with a 20-fold molar excess of the more physiologically relevant oxidant H_2_O_2_ [27]. Because H_2_O_2_-treated PKA-Cα could be glutathionylated, it is likely that the enzyme was sulfenylated by H_2_O_2_ under the experimental conditions. Likewise, pre-incubation with H_2_O_2_ also altered PKA-Cα’s activity toward several substrates (please see below). Thus, at least at a relatively low 20:1 H_2_O_2_:PKA-Cα molar ratio, PKA-Cα can be sulfenylated without promoting disulfide bond formation. Because sulfenylation (as well as subsequent sulfinylation and sulfonylation) is expected to alter the size and physiochemical properties of PKA-Cα^C199^, this may represent an alternative mode by which redox modification of PKA-Cα regulates the catalytic subunit’s function. Indeed, the presence of PKA-Cα^C199^ in the P+1 loop, coupled with its sensitivity to oxidation, makes this residue an attractive candidate for redox-dependent regulation. For example, the P+1 loop is known to play an important role in substrate recognition, where it forms a hydrophobic docking site for non-polar residues found in the P+1 position of many PKA substrates [237,238,239]. Likewise, the P+1 loop helps orient the phosphoacceptor site for nucleophilic attack by the γ-phosphate of ATP during catalysis [237]. Consequently, redox modification of C199 has the potential to alter interactions between PKA-Cα and its substrates. For instance, we found that oxidation of PKA-Cα by low levels of H_2_O_2_ enhanced PKA-mediated phosphorylation of Kemptide, CREBtide, and Crosstide to a similar extent [27]. However, the biochemical mechanisms underlying these changes appear to vary depending on the substrate under study. For instance, H_2_O_2_-dependent oxidation of PKA-Cα caused an approximately two-fold increase in its affinity for Kemptide. In contrast, the affinity of oxidized PKA-Cα for CREBtide *decreased* by approximately 2.5-fold, while that for Crosstide only changed marginally. In the case of CREBtide, kinetic analysis using surface plasmon resonance (SPR) spectroscopy suggested that the oxidation-dependent decrease in affinity was driven largely by an approximately three-fold increase in the apparent off-rate (*k_off_*_,*app*_). This was coupled with a modest (20%) increase in the apparent on-rate (*k_on_*_,*app*_). Intriguingly, at higher concentrations of H_2_O_2_, we observed differential changes in activity toward these substrates. For instance, PKA-Cα’s activity toward Kemptide and Crosstide decreased below baseline at H_2_O_2_:PKA-Cα molar ratios of 60:1 and 80:1, respectively, while its activity toward CREBtide remained high, even at the highest molar ratio in the assay (corresponding to a 160-fold molar excess of H_2_O_2_ over PKA-Cα). Importantly, no H_2_O_2_-dependent changes in PKA-Cα activity were observed for any of the substrates when PKA-Cα(C199S) or PKA-Cα(C199A) were substituted for the wild-type enzyme, suggesting that the observed H_2_O_2_-dependent changes in activity were due to redox modification of PKA-Cα^C199^.

The observed decrease in PKA-Cα activity toward Kemptide at high concentrations of H_2_O_2_ is consistent with a recent report by Byrne et al., who observed a dose-dependent decrease in PKA-Cα activity toward Kemptide following incubation with various concentrations of H_2_O_2_ [22]. While the molar ratios of H_2_O_2_:PKA-Cα used in these studies were considerably higher than those used during our experiments, they support the notion that H_2_O_2_-dependent oxidation can alter PKA-Cα activity toward its substrates. In the future, it will be interesting to see whether PKA-Cα forms intra- and/or intermolecular crosslinks at the higher H_2_O_2_ concentrations used by Byrne and colleagues, similar to what was observed following diamide-mediated oxidation (Figure 7) [27,30]. Regardless, taken in aggregate, these studies suggest that direct modification of PKA-Cα^C199^ by the physiological oxidant H_2_O_2_ can alter PKA-Cα’s interactions with its substrates. Intriguingly, the magnitude and direction of these changes may be dependent on the specific substrate under study. Indeed, these studies are consistent with a model where oxidation-induced changes in the size and physiochemical properties of C199 differentially alter PKA-Cα’s ability to interact with its substrates (Figure 7). For example, interactions between PKA-Cα and substrates containing a bulky, hydrophobic residue(s) in the vicinity of the P+1 loop may be disrupted when PKA-Cα^C199^ is oxidized by H_2_O_2_. Alternatively, substrates that contain a positively charged residue(s) in the analogous position may form H-bonds or salt bridges with -SOH (or higher order oxoforms), thereby strengthening interactions with oxidized PKA-Cα. Finally, still other substrates, such as those with small aliphatic residues in close proximity to oxidized C199, may not undergo much change at all. Such a mechanism could potentially lead to different substrate profiles in the oxidized and reduced states. In support of this notion, in the macrophage hypoxia study described earlier, Srinivasan et al. found that distinct sites on cytochrome c oxidase (CcO) are phosphorylated by PKA during hypoxia and normoxia, suggesting that redox modification may even alter site selection within the same substrate [218]. Similarly, other types of redox modifications, such as glutathionylation or redox-active electrophilic lipids, could alter PKA-Cα’s substrate interactions in a similar manner, as described below.

In addition to the direct modulation of PKA-Cα activity alluded to earlier, diamide-dependent oxidation of C199 has also been shown to promote the formation of a mixed disulfide with glutathione [30,240,241]. Likewise, as alluded to above, we demonstrated that PKA-Cα oxidized by H_2_O_2_ can be glutathionylated [27]. Despite its relatively large size, molecular modeling suggests that the bulky glutathione moiety is well accommodated in PKA-Cα’s active site (our unpublished data). Thus, aside from its role in protecting PKA-Cα from hyperoxidation, glutathionylation may also serve as a bona fide oxPTM. For instance, previous studies demonstrated that glutathionylation of PKA-Cα decreased its activity toward Kemptide nearly ten-fold [30]. This effect, which was abrogated in the presence of DTT, was completely reversed by treatment of glutathionylated PKA-Cα with the thioltransferase glutaredoxin. Likewise, mutation of C199 to Ala prevented glutathione-mediated inhibition of the enzyme. Together, these data suggest that the bulky glutathione moiety disrupts interactions between PKA-Cα and Kemptide. In the future, it will be interesting to determine whether other substrates are affected in a similar manner or if gluthionylation-dependent changes in PKA-Cα activity are substrate-specific. Interestingly, glutathionylation of PKA-Cα^C199^ may also regulate its activity in other ways, as outlined below.

Many kinases, including those in the AGC family, are themselves regulated by phosphorylation [242]. Although many protein kinases are triggered by transient phosphorylation of their activation loops, PKA-Cα appears to be phosphorylated co-translationally by either phosphatidylinositide-dependent kinase 1 (PDK1) or other PKA-Cα molecules, leading to a constitutively active form whose activity is regulated predominantly by binding R subunits [243,244,245]. PKA-Cα has two main phosphorylation sites, one in the activation loop at T197 and the other near the C-terminus at S338 [243]. The phosphorylation of T197 is important for activation because the phosphate moiety is required to coordinate critical catalytic residues in the G-loop involved in ATP binding, leading to efficient enzyme activity [246,247]. Because this residue is constitutively phosphorylated and resistant to phosphatase treatment, dephosphorylation of T197 has not been extensively recognized as a regulatory mechanism [243,248,249]. However, glutathionylation of C199, which lies just two residues away from T197 in the activation loop, renders T197 more susceptible to dephosphorylation, presumably by a protein phosphatase 2A (PP2A)-like enzyme [241,250,251]. As a result, glutathionylation may promote PP2A-mediated inhibition of PKA-Cα. Consistent with this notion, Humphries et al. found that diamide-mediated oxidation of PKA-Cα resulted in enhanced dephosphorylation of T197 [241]. Importantly, PDK1 can rephosphorylate the dephosphorylated kinase [252,253,254], restoring its function and providing a reversible mode of regulation.

## 12. Conclusions and Future Perspectives

Even though PKA is one of the most extensively studied eukaryotic protein kinases, critical knowledge gaps remain regarding the impact of oxPTMs on several aspects of its regulation, including holoenzyme conformation, kinase activation, substrate selection, and broader cellular functions. Likewise, the interplay between redox-dependent PKA regulation and classical cAMP-mediated activation is only beginning to be understood, yet this interplay is crucial for determining the overall effect of redox regulation on PKA function in several important cellular contexts. The susceptibility of both PKA regulatory and catalytic subunits to redox modification suggests a complex regulatory network that influences phosphorylation events underlying critical cellular activities, including cardiac contractility, lipogenesis, and inflammation. Therefore, while we begin to gain important insights into the mechanisms of crosstalk between redox- and PKA-dependent signaling, further research is needed to elucidate the physiological and pathological consequences of PKA oxidation. A deeper understanding of these mechanisms may offer new therapeutic targets for oxidative dysregulation of PKA in a variety of pervasive disorders, including cardiovascular disease, diabetes, and various neurological disorders.

## Data Availability

No new data were created or analyzed in this study. Data sharing is not applicable to this article.

## Supplementary Materials

The following supporting information can be downloaded at: https://www.mdpi.com/article/10.3390/life15040655/s1, Table S1: Select search terms used for literature review.; Table S2: Select physiological roles of PKA and associated disorders.

## Abbreviations

The following abbreviations are used in this manuscript:

cAMP	3′,5′-cyclic adenosine monophosphate
PKA	cAMP-dependent protein kinase
PKA-R	PKA regulatory subunit
PKA-C	PKA catalytic subunit
CBD	cAMP binding domain
RS	Recognition sequence
AC	Adenylyl cyclase
PDE	Phosphodiesterase
GPCR	G protein-coupled receptor
AKAP	A-kinase anchoring protein
SR	Sarcoplasmic reticulum
AD	Alzheimer’s disease
PD	Parkinson’s disease
ROS	Reactive oxygen species
O_2_^−^	Superoxide
H_2_O_2_	Hydrogen peroxide
R-SOH	Sulfenic acid
R-SO_2_H	Sulfinic acid
R-SO_3_H	Sulfonic acid
GSH	Reduced glutathione
GSSG	Oxidized glutathione (glutathione disulfide)
SOD	Superoxide dismutase
oxPTM	Oxidative posttranslational modification
PDK1	Phosphoinositide-dependent kinase
DTT	Dithiothreotol
