# Supplementary material for: Redox Regulation of cAMP-Dependent Protein Kinase and Its Role in Health and Disease
Source: Life (Basel). 2025 Apr 16;15(4):655. doi: 10.3390/life15040655 (PMC12029036; doi:10.3390/life15040655)
Supplement: Supplementary file 1 [file life-15-00655-s001.zip › life-3543161-supplementary.pdf]

Supplementary Materials

Table S1. Select search terms used for literature review.

"PKA oxidation"	"Kinase redox regulation"
"PKA signaling"	"PKA and redox signaling"
"PKA activation"	"PKA substrate selection"
"Protein oxidation"	"PKA regulation"
"Kinase oxidation"	"Redox signaling"
"PKA role in [disease]" where the specific disease was specified (e.g., diabetes, cancer, cardiovascular disease, etc.	"Oxidation in [disease]", where the specific disease was specified (e.g., diabetes, cancer, cardiovascular disease, etc.)

Table S2. Select physiological roles of PKA and associated disorders.

Physiological Role PKA	Mechanism/Target	Impact on Function	Impact of PKA Dysregulation on Disease Etiology	References
**Cardiovascular System**				
Regulation of Cardiac Contractility	Phosphorylation of L-type Ca^{2+} channel (CaV1.2) via Rad at Ser25, Ser38, Ser272, and Ser300	Enhances Ca^{2+} influx, increases channel open probability, and contributes to positive inotropic response	Altered PKA-Cα expression/activity leads to impaired contractility, contributing to heart failure and pathological remodeling	[44, 111, 104]
Sarcoplasmic Reticulum (SR) Ca^{2+} Handling	Phosphorylation of phospholamban (PLN) at Ser16	Disrupts PLN-SERCA2 interaction, accelerates Ca^{2+} reuptake into SR, and enhances myocardial relaxation (positive lusitropy)	Dysregulated PKA-PLN interaction leads to impaired Ca^{2+} reuptake, arrhythmias, and contractile dysfunction	[112-114
Regulation of Ca^{2+} dynamics through the Ryanodine Receptor 2 (RyR2)	Phosphorylation of RyR2	Enhances Ca^{2+} release during systole, strengthening contractility	Dysfunctional PKA-RyR2 regulation causes Ca^{2+} leakage, contributing to arrhythmias and heart failure	[110]
Modulation of Myofilament Dynamics	Phosphorylation of cardiac myosin-binding protein C	Enhances actin-myosin interaction, accelerates cross-	Impaired cMyBP-C phosphorylation decreases	[116-119]

	(cMyBP-C) at cardiac-specific M-motif	bridge cycling, and increases contractile force	contractility, contributing to cardiac dysfunction in heart failure	
Sarcomere Ca^{2+} Sensitivity	Phosphorylation of cardiac troponin I (cTnI) at Ser23/24	Reduces Ca^{2+} affinity of troponin C (cTnC), improving lusitropy and promoting efficient relaxation	Reduced cTnI phosphorylation leads to impaired relaxation and diastolic dysfunction in heart failure	[120, 121, 126-128]
Cardiac Remodeling	Altered PKA-C subunits	Promotes pathological cardiac remodeling and contractile dysfunction	Altered PKA-Cα expression/activity linked to hypertrophy, heart failure, and impaired myocardial contractility	[104]
Vascular Tone and Hypertension	Dysregulated PKA signaling in vascular smooth muscle cells	Maintains vascular tone by reducing vasoconstriction	Dysregulated PKA causes increased vasoconstriction and elevated blood pressure, leading to hypertension	[102, 103]
**Metabolic Regulation**				
Regulation of Glucose and Lipid Metabolism	PKA activity in adipocytes and hepatocytes regulates glucose uptake, lipid breakdown, and insulin sensitivity	Maintains metabolic homeostasis by promoting balanced glucose and lipid metabolism	Dysregulated PKA-Cα activity in adipocytes impairs insulin signaling, contributing to insulin resistance and type 2 diabetes	[129, 130]
Adipogenesis and Insulin Sensitivity	PKA-Cα signaling in adipocytes	Regulates adipogenesis and promotes insulin sensitivity	Increased PKA-Cα activity leads to impaired adipogenesis and insulin resistance, driving the development of metabolic syndrome	[130]
Control of Liver Glucose and Lipid Homeostasis	PKA signaling in hepatocytes	Regulates hepatic glucose production and lipid metabolism	Dysregulated PKA activity in the liver contributes to abnormal	[131]

			glucose/lipid metabolism and progression of non-alcoholic fatty liver disease (NAFLD)	
Regulation of lipid metabolism and storage	PKA-dependent regulation of lipid breakdown and storage	Maintains normal lipid storage and prevents excessive fat accumulation in the liver	Impaired PKA signaling exacerbates NAFLD, leading to liver dysfunction, inflammation, and insulin resistance	[131]
Genetic Regulation in Rare Disorders	Mutations in PKA regulatory (R) subunits	Maintain appropriate PKA activation through balanced regulation of PKA-C subunits	Mutations in PKA-R subunits are linked to rare metabolic disorders like Carney syndrome, which affects multiple organ systems	[132, 133
Regulation of Lipolysis and Insulin Signaling	PKA-mediated signaling in adipose tissue	Controls lipolysis and insulin signaling	Dysregulated PKA leads to increased lipolysis, dyslipidemia, and insulin resistance, contributing to diabetes and obesity.	[129]
**Central nervous system**				
Synaptic Plasticity	PKA-mediated phosphorylation of synaptic proteins	Enhances long-term potentiation (LTP) and synaptic strength	Aberrant PKA-Cγ activation leads to synaptic dysfunction, contributing to cognitive deficits in Alzheimer's disease (AD)	[134, 135]
Neuronal Survival	PKA-dependent regulation of pro-survival signaling pathways	Promotes neuronal viability and reduces apoptosis	Dysregulated PKA activity can trigger neuronal apoptosis, contributing to neurodegeneration in AD and Parkinson’s disease (PD)	[136, 138]

Neurotransmitter Release	PKA-mediated phosphorylation of ion channels and vesicle-associated proteins	Facilitates dopamine and release of other neurotransmitters	Impaired PKA signaling in dopaminergic neurons leads to disrupted dopamine release, contributing to PD	[136, 137]
Cognitive Function	PKA involvement in memory formation and synaptic remodeling	Supports learning and memory by enhancing synaptic connections	Dysregulated PKA signaling leads to synaptic dysfunction, memory impairment, and cognitive decline	[135, 136]
Dopamine Signaling	PKA regulation of dopamine signaling and neuronal health	Maintains dopaminergic neuron function and dopamine release	Dysregulated PKA in PD exacerbates dopaminergic neuronal loss and motor dysfunction	[136, 137]
Modulating mood	PKA modulation of serotonin and dopamine signaling	Regulates mood and emotional stability	Impaired PKA activity is linked to serotonin/dopamine imbalance, contributing to mood disorders like depression	[138]
Amyloid Precursor protein (APP) Metabolism	PKA regulation of amyloid precursor protein (APP) processing and synaptic health	Protects against amyloid-beta accumulation and neurodegeneration	Aberrant PKA activation contributes to amyloid toxicity, synaptic loss, and neuronal death in AD and PD	[135]
Cancer Biology				
Cell Proliferation and Survival	PKA-Cα promotes phosphorylation of cell cycle regulators and survival pathways.	Enhance cell proliferation and survival.	Elevated PKA-Cα expression is linked to increased tumor cell proliferation, migration, and invasion in breast cancer	[44, 140, 141]
Tumor suppression	PKA-Cβ modulation of signaling pathways	Reduces tumor growth and prevents metastasis	Decreased PKA-Cβ expression is associated with colorectal cancer	[142]

	controlling growth and differentiation		progression and metastasis	
Cell Migration	PKA-RIIβ regulation of cytoskeletal dynamics and cell adhesion	Reduces invasiveness and metastasis	Downregulation of PKA-RIIβ linked to enhanced invasiveness and metastasis in melanoma	[44]
Oncogenic Mutations in PKA Subunits	Mutations in PKA-C and PKA-R genes affecting kinase activity	Can either enhance or suppress tumor growth, depending on cellular context	Mutations in PKA subunit genes contribute to various cancers, emphasizing their role in tumorigenesis	[44, 143]
Regulation of Apoptosis	PKA-RIα regulation of proliferative and apoptotic signaling pathways	Promotes tumor growth when PKA-RIα is overexpressed	Aberrant expression of PKA-RIα associated with increased cell proliferation and tumor growth in breast cancer	[44, 144, 46]
Regulation of Cell Cycle Progression and Differentiation	PKA-Cβ modulation of cell cycle and differentiation pathways	Suppresses tumor progression	Loss of PKA-Cβ expression linked to increased aggressiveness of colorectal cancer and metastasis	[142]
